# Outcomes of Patients With Chronic Lymphocytic Leukemia Treated With Bruton Tyrosine Kinase Inhibitors (BTKi): A Retrospective Study From a Tertiary Healthcare Center in North India

**DOI:** 10.7759/cureus.117629

**Published:** 2026-10-08

**Authors:** Alpika Shukla, Amit Pandey, Shailendra P Verma, Swasti Sinha, Anil Tripathi, Neha Shukla, Gaurav Dutta, P Raghuveer

**Affiliations:** 1 Hematology and Oncology/Clinical Hematology, King George's Medical University, Lucknow, IND; 2 Medical Oncology, Dr. Ram Manohar Lohia Institute of Medical Sciences, Lucknow, IND; 3 Clinical Hematology, King George's Medical University, Lucknow, IND; 4 Hematology and Oncology/Clinical Hematology, Hind Institute of Medical Sciences, Lucknow, IND; 5 Statistics, University of Lucknow, Lucknow, IND

**Keywords:** acalabrutinib, bruton tyrosine kinase (btk) inhibitor, chronic lymphocytic leukemia (cll), ibrutinib therapy, overall survival (os), progression-free survival

## Abstract

Background and objectives: Bruton tyrosine kinase inhibitors (BTKi) are a pivotal advancement in treating chronic lymphocytic leukemia (CLL). However, their adoption faces challenges such as high discontinuation rates caused by adverse events like cardiac and hematologic toxicities and a significant financial burden. Our study aims to evaluate the clinical spectrum and outcomes of patients with CLL receiving BTKi therapy in India. The objectives are to evaluate progression-free survival and overall survival in CLL patients on BTKi and to assess the BTKi toxicity profile.

Materials and methods: This retrospective, single-center study analyzed data from 41 patients with CLL treated with BTKi between January 2019 and November 2024.

Results: Twenty-five male patients and 16 female patients were evaluated, with a median age of 64 years (range 42-90). Twelve patients (29.2%) had no cytogenetic abnormalities, while 29 (70.7%) had at least one abnormality; 13q deletion was the most common. BTKi was used as first-line therapy in 29 patients (70.7%), with acalabrutinib being the most commonly used agent, administered to 17 (58.6%) patients. Cardiac events were observed in 8 (19.5%) patients, including hypertension in four (9.7%) and atrial fibrillation in two (4.9%). Gastrointestinal adverse events were noted in 7 (17%) patients. One patient had Richter transformation to diffuse large B-cell lymphoma. At a median follow-up of 24 months, progression-free survival was 80% and overall survival was 95%.

Conclusions: BTKi therapy is effective and well tolerated in Indian CLL patients, but early discontinuation highlights the need for improved adherence strategies.

## Introduction

Bruton tyrosine kinase (BTK) is a crucial enzyme in the B-cell receptor (BCR) signaling pathway. It plays an important role in the survival and proliferation of malignant B-cells in chronic lymphocytic leukemia (CLL) [1]. Inhibiting BTK disrupts BCR signaling, interfering with pathways essential for CLL cell growth and survival [2,3].

Ibrutinib was the first-in-class BTK inhibitor (BTKi) approved for CLL and has since become an important component of treatment for both previously treated and untreated patients, including those with high-risk disease [4]. Acalabrutinib is a second-generation BTKi with greater selectivity for BTK and has shown efficacy in CLL with a potentially more favorable toxicity profile than ibrutinib.

Common adverse effects associated with BTKi therapy include bleeding, infections, atrial fibrillation, diarrhea, fatigue, rash, hypertension, and other cardiovascular complications. Bleeding is partly related to inhibition of platelet signaling and function. Second-generation BTKi such as acalabrutinib may have a more favorable cardiovascular and bleeding profile than ibrutinib.

BTKi therapy is generally administered continuously until disease progression or unacceptable toxicity. Selection among ibrutinib, acalabrutinib, and other BTKi may depend on patient-specific factors, including age, comorbidities, cardiovascular risk, previous treatment, toxicity profile, availability, and affordability. BTK inhibition has also become particularly important in patients with high-risk biological features such as del(17p) and TP53 abnormalities.

In India, access to targeted therapies has increased, although affordability, availability, reimbursement, and treatment adherence continue to influence the use of novel agents. There has been a clear shift toward non-chemotherapy-based treatment approaches in CLL, particularly with increasing availability of targeted therapies. Indian real-world data regarding BTKi treatment remain relatively limited.

This study aimed to evaluate the clinical profile of patients with CLL treated with ibrutinib or acalabrutinib, assess the toxicity profile of these drugs, and evaluate survival outcomes to better understand the role of BTKi in the long-term management of CLL in an Indian real-world setting.

## Materials and methods

This retrospective, single-center observational study was conducted at the Department of Clinical Hematology of a tertiary healthcare center. The study included patients with CLL who received treatment with a BTKi between January 2019 and November 2024.

Clinical and treatment-related data were retrospectively retrieved from the department's medical records. Patients were eligible for inclusion if they had a confirmed diagnosis of CLL, received a BTKi (ibrutinib or acalabrutinib), and completed at least six months of BTKi therapy. Patients who had received less than six months of BTKi treatment were excluded from the analysis.

The collected data included demographic and clinical characteristics, disease-related features, BTKi type, treatment duration, and treatment-related adverse events. Particular attention was given to clinically relevant toxicities associated with BTKi therapy, including bleeding, infections, atrial fibrillation, hypertension, gastrointestinal adverse events, and other cardiovascular or treatment-related complications. Data on treatment response, disease progression, treatment discontinuation, and survival outcomes were also assessed where available.

Patients were categorized according to the BTKi received (ibrutinib or acalabrutinib), and clinical characteristics, toxicity profiles, and treatment outcomes were compared between the treatment groups.

Categorical variables were expressed as frequency (n) and percentage (%). For continuous variables, the Student's t-test or Mann-Whitney U test was used, as appropriate, based on the distribution of the data. Survival outcomes were estimated using the Kaplan-Meier method. All statistical tests were two-sided, and p-values <0.05 were considered statistically significant. Statistical analyses were performed using SPSS Statistics version 29.0 (IBM Corp. Released 2022. IBM SPSS Statistics for Windows, Version 29.0. Armonk, NY).

The primary objective was to evaluate the clinical profile and treatment-related toxicity of patients with CLL receiving BTKi therapy. Secondary objectives included assessment of treatment outcomes, treatment discontinuation, and survival in this real-world cohort.

## Results

Of the 41 patients studied, 25 were male, and 16 were female, with a median age of 64 years (Table 1).

**Table 1:** Baseline clinical characteristics of the study population (N = 41) LDH: lactate dehydrogenase

Characteristic	n	Percentage (%)
Age		
<65 years	18	43.9
≥65 years	23	56.1
Sex		
Female	16	39.02
Male	25	60.98
LDH		
<450	16	39.02
≥450	25	60.98
Coombs test		
Positive	3	7.32
Negative	34	82.93
Not available/not performed	4	9.76

Cytogenetic analysis revealed that 12 patients (29.2%) had no cytogenetic abnormalities, while the remaining 70.7% exhibited at least one abnormality (Figure 1). The most common cytogenetic abnormality was the 13q deletion. IGHV mutations were detected in four out of 41 patients.

**Figure 1:**
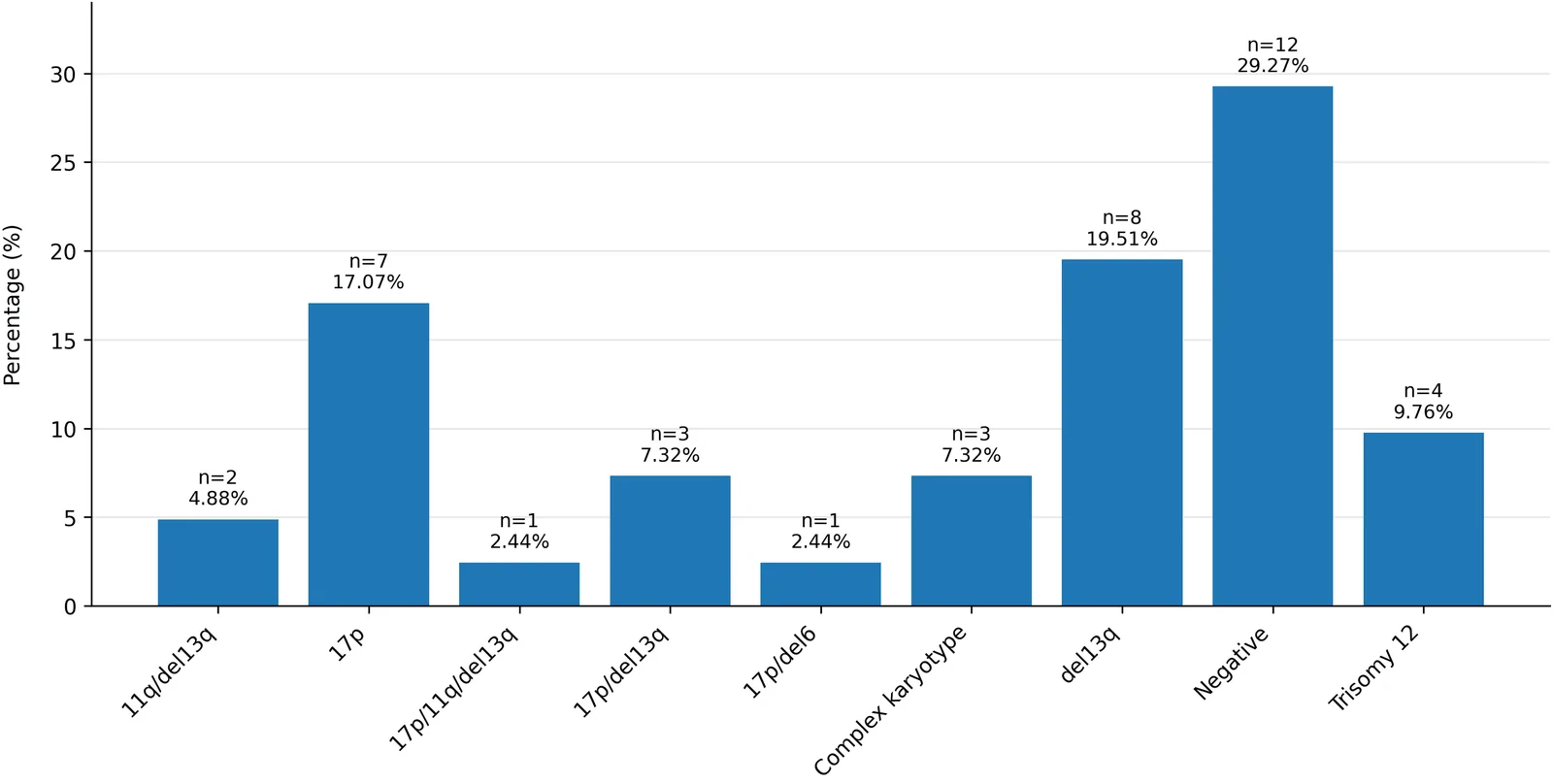
Karyotyping and FISH FISH: fluorescence in situ hybridization, del: deletion, 11q: long arm of chromosome 11, 13q: long arm of chromosome 13, 17p: short arm of chromosome 17

For treatment, BTKi were used as first-line therapy in 29 patients (70.7%). Acalabrutinib was the most frequently used BTKi, administered to 17 patients (58.6%). Additionally, 12 (29.2%) patients received BTKi treatment in the relapsed or refractory setting. In the relapse/refractory setting, two-thirds of patients received ibrutinib, whereas one-third received acalabrutinib (Figures 2-3).

**Figure 2:**
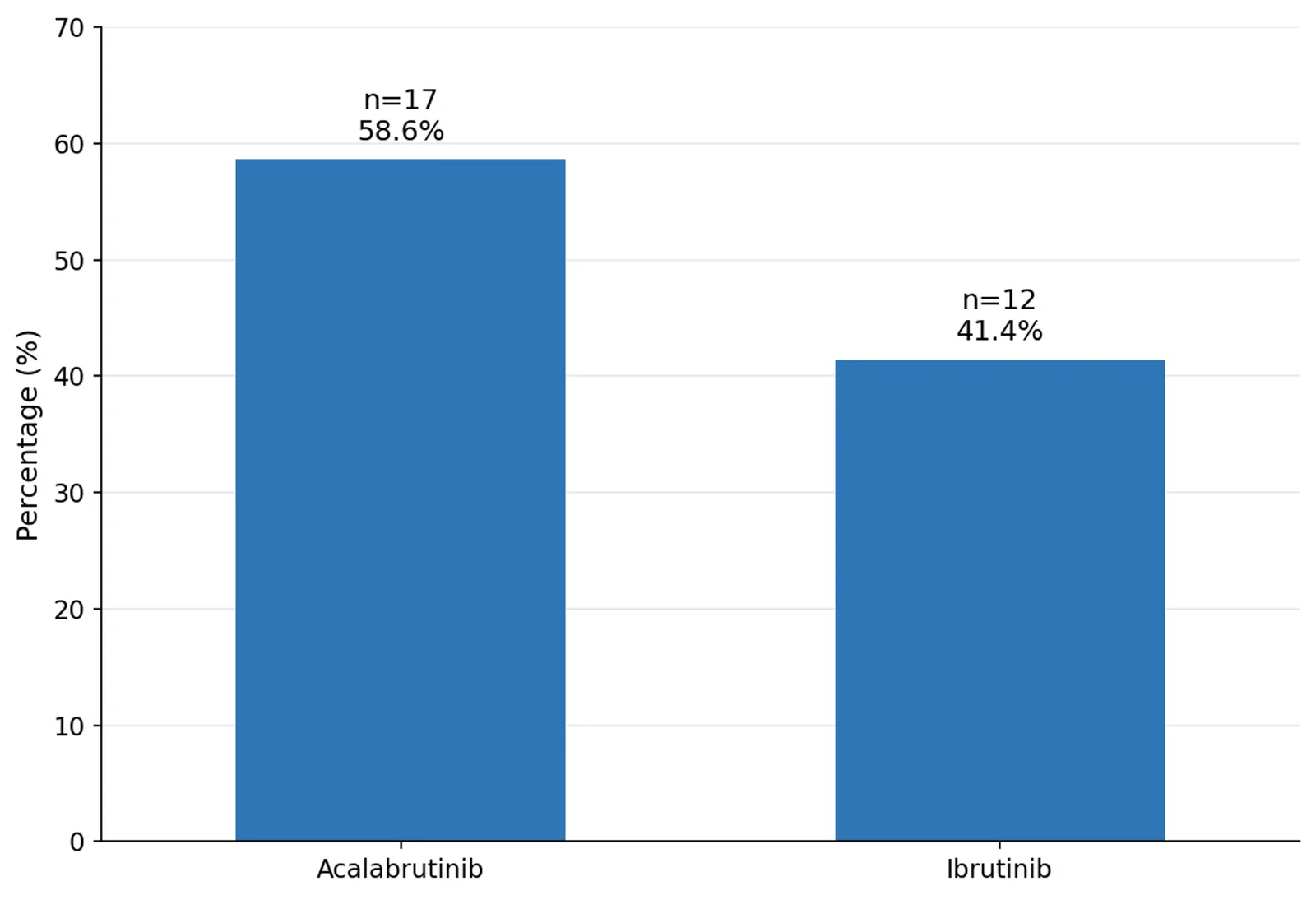
BTKi as first-line therapy BTKi: Bruton tyrosine kinase inhibitor

**Figure 3:**
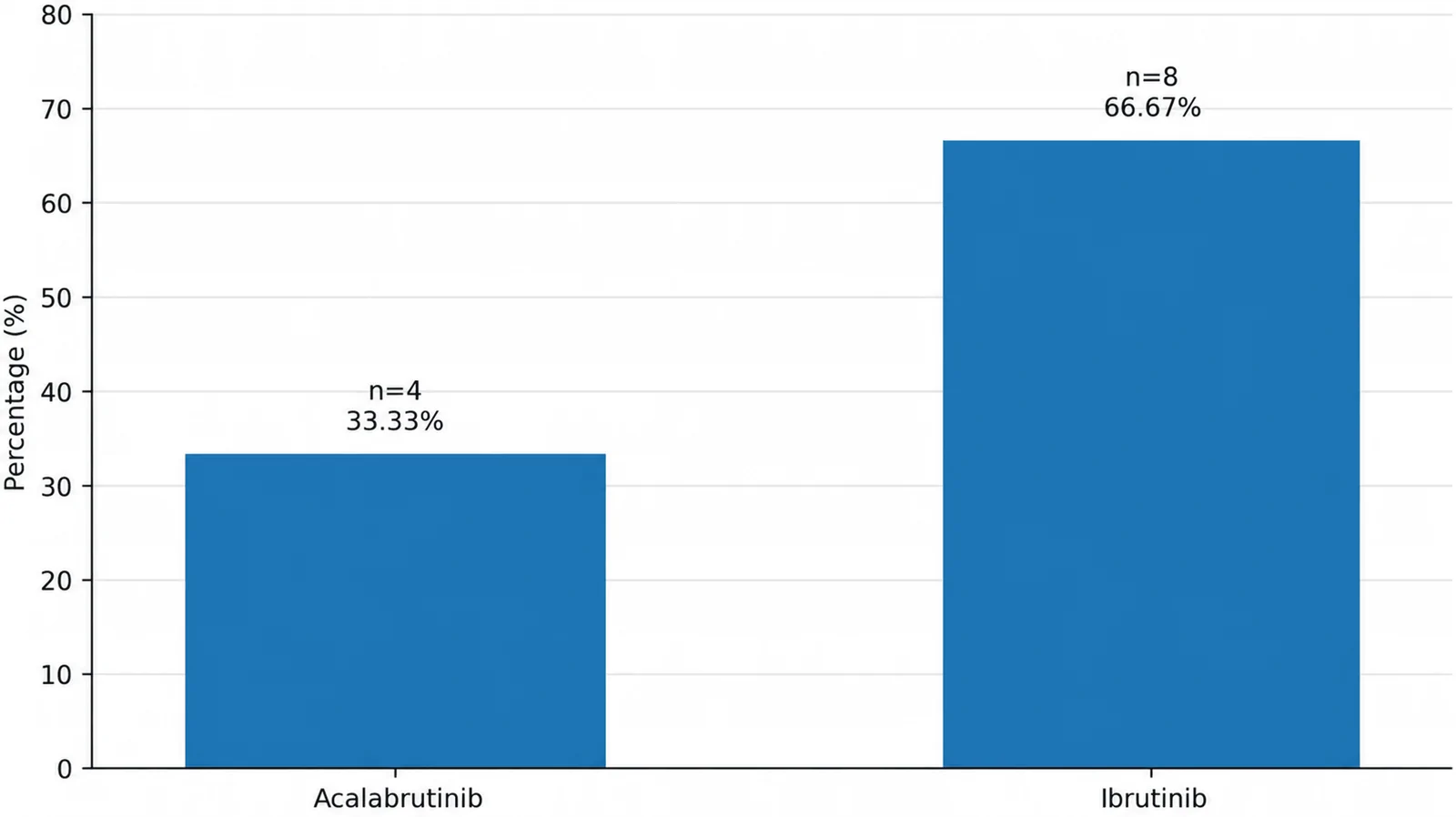
BTKi as second-line therapy BTKi: Bruton tyrosine kinase inhibitor

The study also evaluated the time to resolution of lymphocytosis in patients with CLL receiving BTKi therapy. The median time to resolution was nine months for patients treated with acalabrutinib and 12 months for those treated with ibrutinib.

Diabetes was the most frequently observed comorbidity, found in three patients (7.3%), followed by hypertension in two patients (4.9%). Most patients (31; 75.6%) had no comorbid conditions (Figure 4).

**Figure 4:**
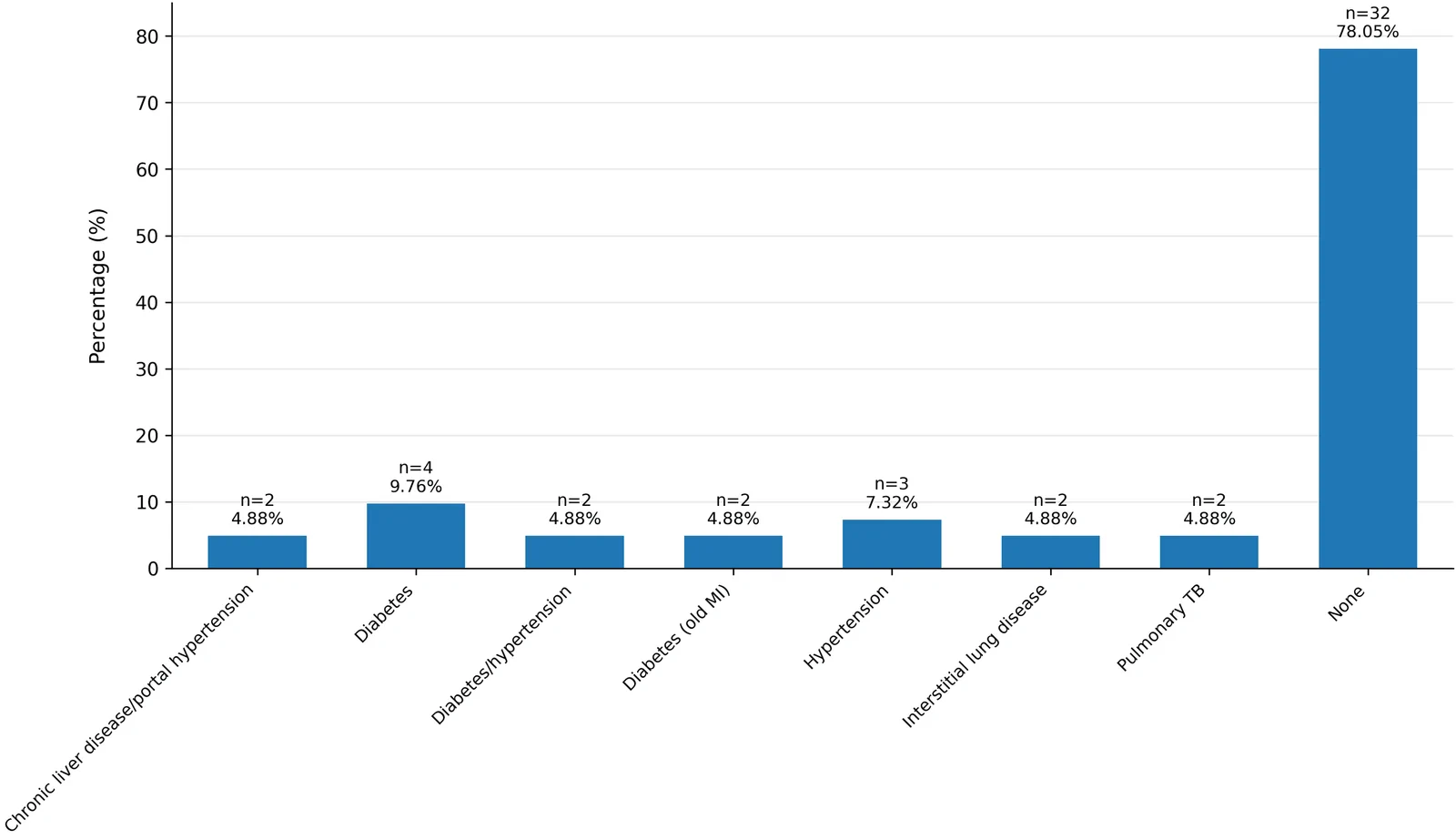
Comorbid conditions at baseline TB: tuberculosis

Regarding adverse events, cardiac complications were observed in eight patients (19.5%), including hypertension in four patients (9.7%), atrial fibrillation in two patients (4.9%), and cardiac failure in two patients (4.9%). Seven (17%) patients reported fatigue, whereas three patients experienced headaches during BTKi treatment. Additionally, one patient (2.4%) experienced per-rectal bleeding, and two patients (4.9%) experienced occasional bruising. Gastrointestinal side effects, such as nausea and diarrhea, were noted in seven patients (17%), with one case (2.4%) of grade 3 diarrhea. Respiratory infections, specifically lower respiratory tract infections, were documented in four patients (9.7%) (Figure 5). One patient developed Richter transformation to diffuse large B-cell lymphoma (DLBCL) and was switched to R-CHOP-based chemotherapy.

**Figure 5:**
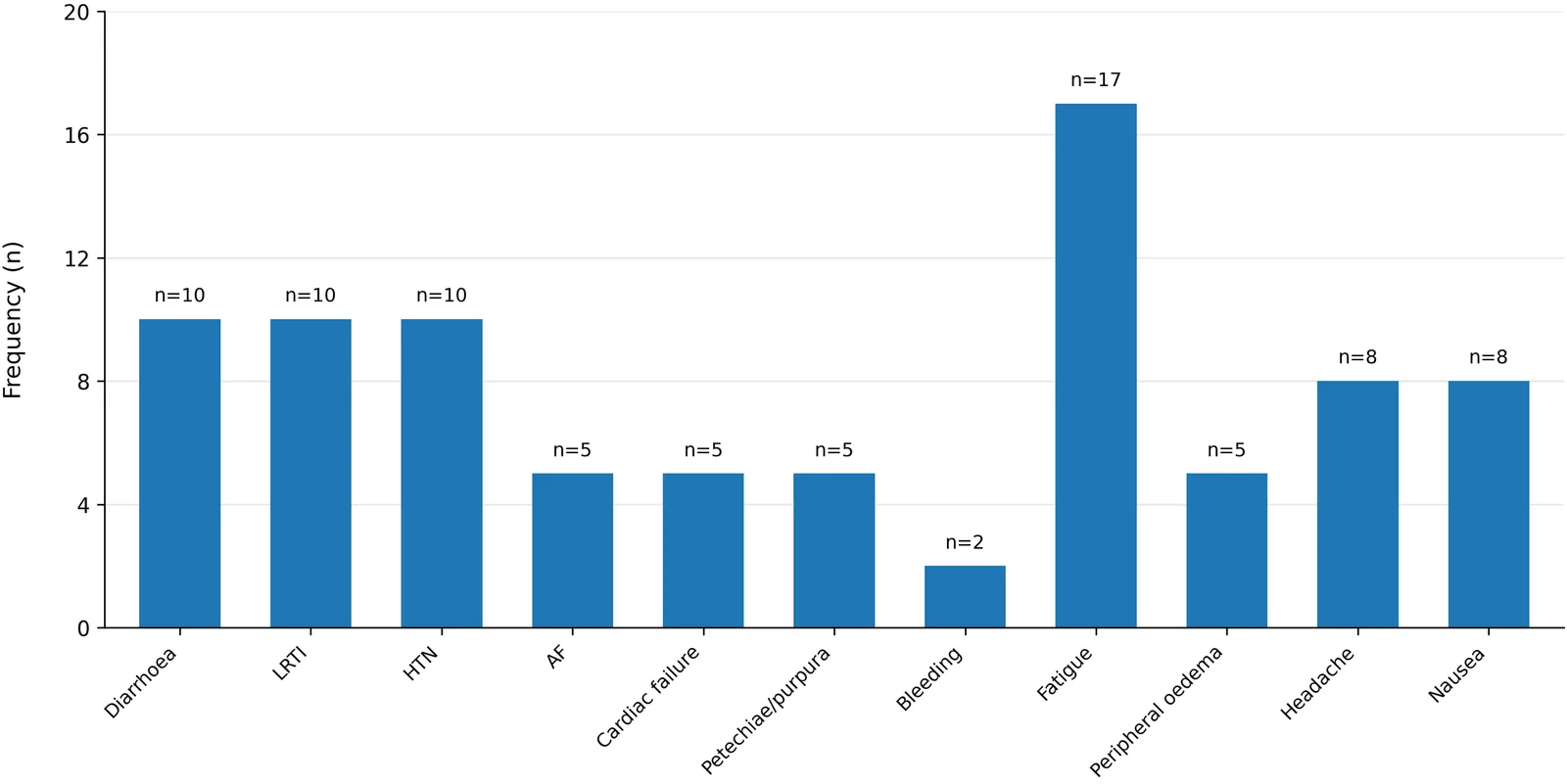
Adverse events during BTKi therapy AF: atrial fibrillation, HTN: hypertension, LRTI: lower respiratory tract infection

Survival analysis comparing patients with and without 17p deletion showed a statistically significant difference (p = 0.014). Median survival was 30 months for patients with 17p deletion, whereas it was not reached for those without 17p deletion (Figure 6).

**Figure 6:**
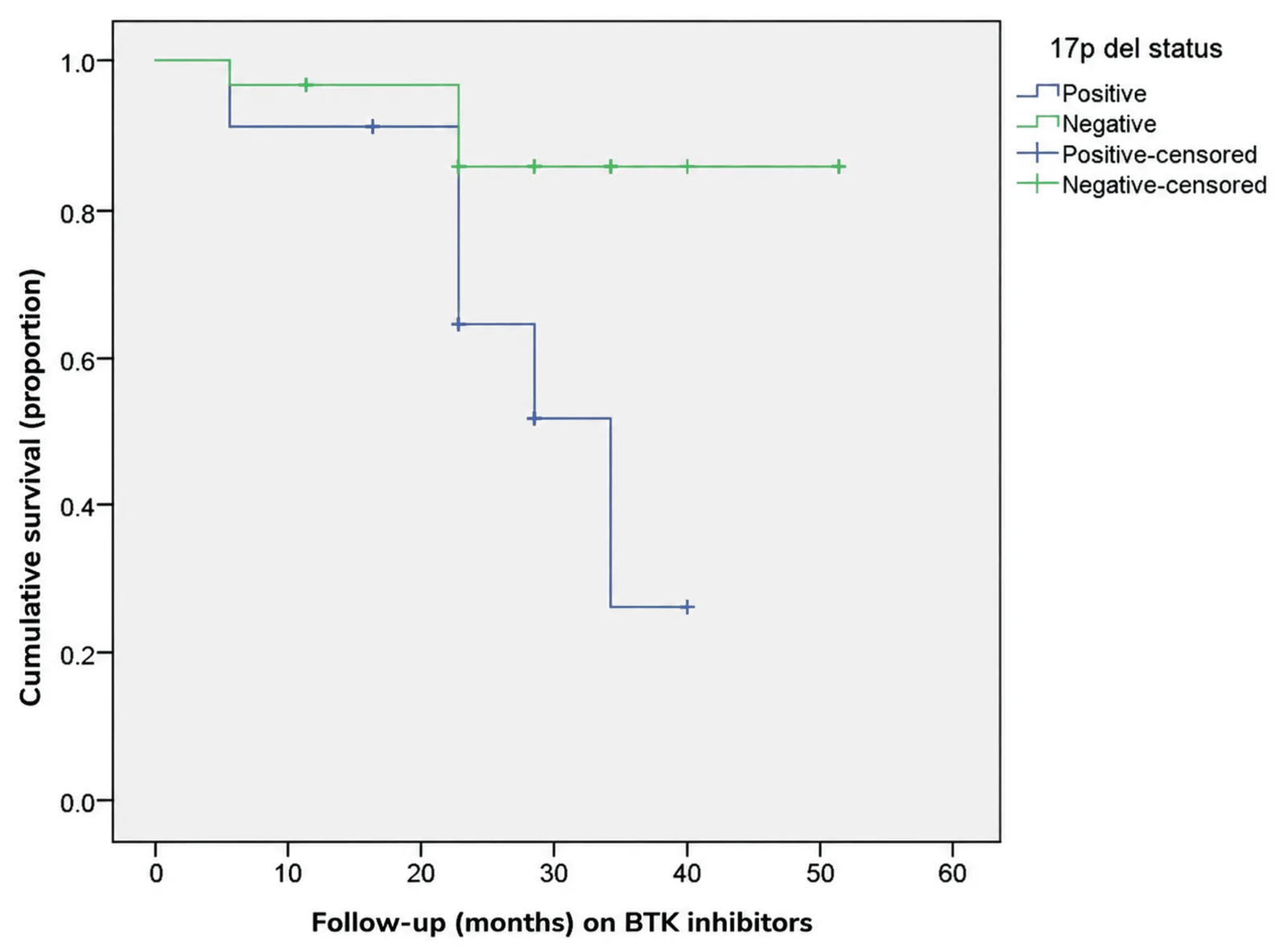
Kaplan-Meier estimates of overall survival according to 17p deletion status 17p: short arm of chromosome 17, del: deletion, BTK: Bruton tyrosine kinase

At a median follow-up of 24 months, the study reported 80% progression-free survival and 95% overall survival (Figures 7-8).

**Figure 7:**
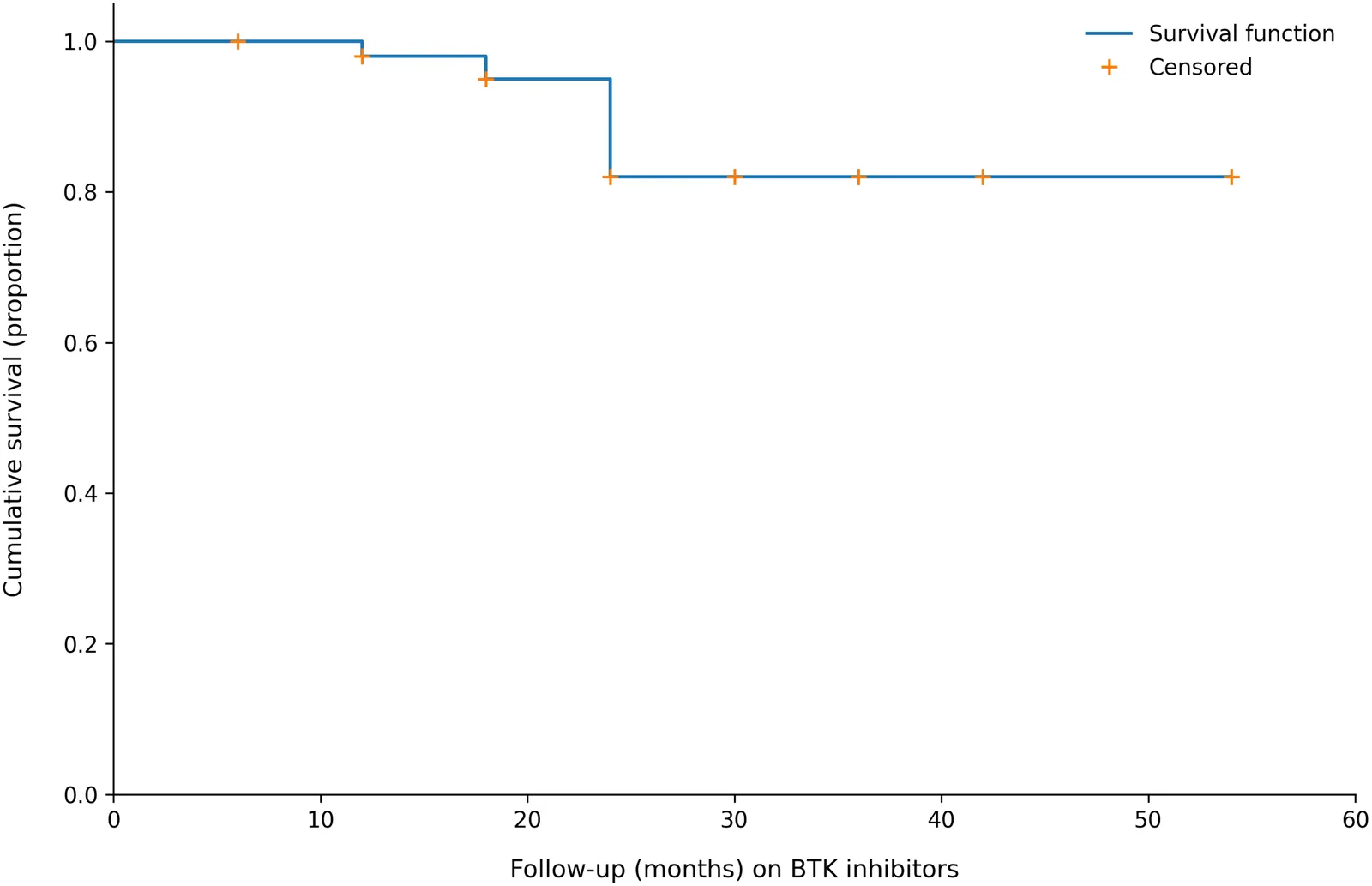
Kaplan-Meier estimates of progression-free survival BTK: Bruton tyrosine kinase

**Figure 8:**
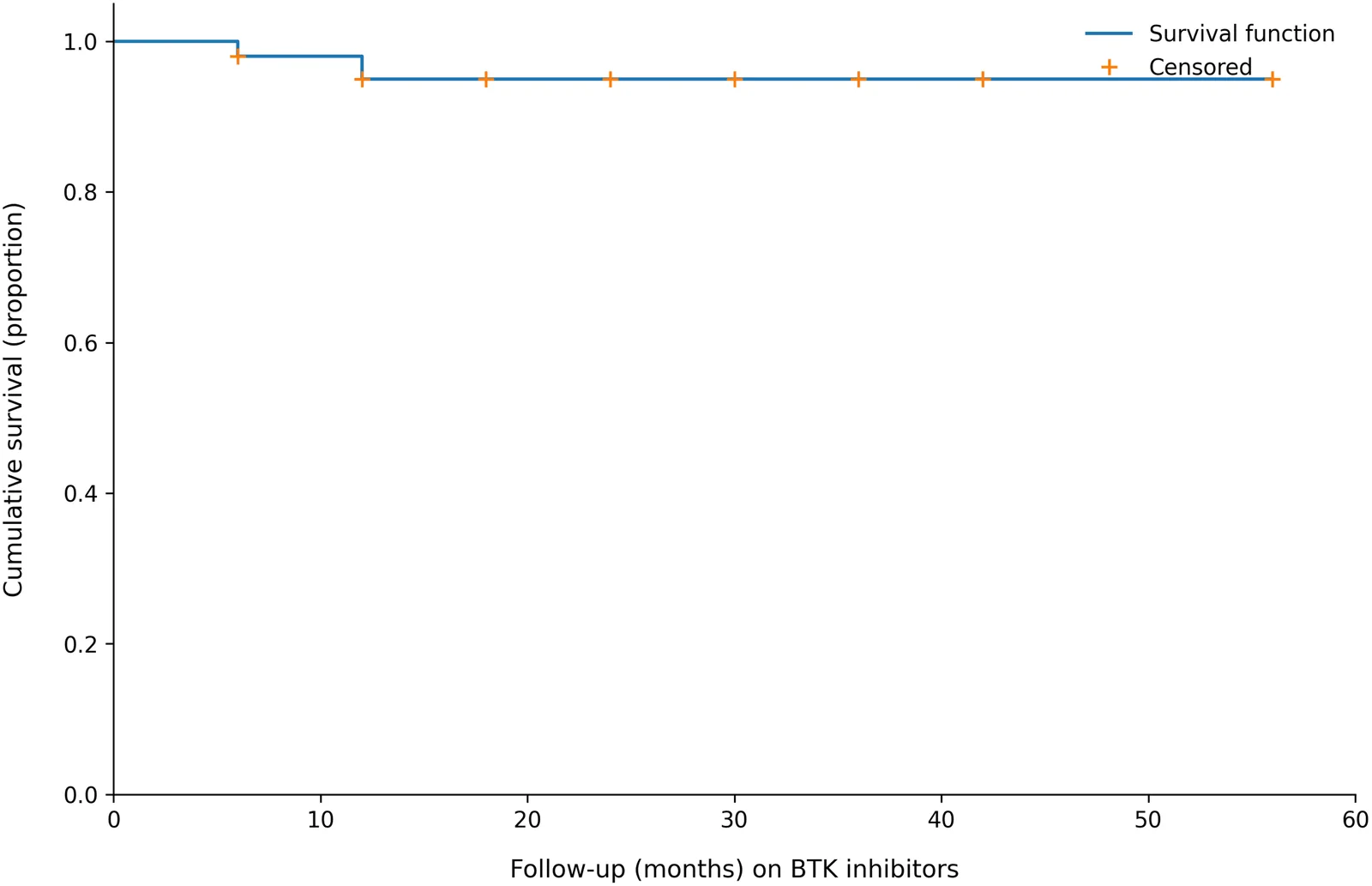
Kaplan-Meier estimates of overall survival BTK: Bruton tyrosine kinase

## Discussion

This study evaluated real-world clinical outcomes in patients with CLL treated with BTKi at a tertiary healthcare center in North India. The study included 41 patients treated between January 2019 and November 2024, with a median age of 64 years. Most patients received BTKi therapy as first-line treatment, and acalabrutinib was the most commonly used agent.

The findings are consistent with the increasing role of BTK inhibition in CLL. BTK is an essential component of BCR signaling, and pharmacological inhibition interferes with signaling pathways involved in malignant B-cell survival and proliferation [1-3]. The clinical development of ibrutinib established BTK inhibition as an effective treatment strategy for CLL. In contrast, subsequent development of more selective agents such as acalabrutinib has aimed to preserve efficacy while reducing off-target toxicity [4].

Acalabrutinib and ibrutinib

Acalabrutinib has emerged as an important alternative to ibrutinib. The ELEVATE-TN and ASCEND trials demonstrated the efficacy of acalabrutinib in treatment-naïve and relapsed/refractory CLL, respectively [5,6]. Specifically, ELEVATE-TN showed sustained efficacy of acalabrutinib-based treatment in previously untreated CLL [5]. In the ASCEND trial, acalabrutinib significantly prolonged progression-free survival compared with investigator's choice of idelalisib plus rituximab or bendamustine plus rituximab [6].

In our cohort, acalabrutinib was the most frequently used BTKi. The median time to resolution of lymphocytosis was nine months with acalabrutinib compared with 12 months with ibrutinib. Redistribution lymphocytosis is a well-recognized pharmacodynamic effect of BTK inhibition. It should be interpreted in the context of other clinical and laboratory findings rather than being considered as isolated evidence of treatment failure.

Bleeding and platelet dysfunction

Bleeding is a recognized complication of BTKi treatment and is thought to result predominantly from platelet dysfunction rather than thrombocytopenia alone. Dmitrieva et al. demonstrated abnormalities in platelet aggregation and function among patients receiving ibrutinib, supporting a platelet-mediated mechanism for bleeding [7].

In our cohort, bleeding events were relatively infrequent. One patient (2.4%) experienced per-rectal bleeding, and two patients (4.9%) reported occasional bruising. The low frequency of clinically significant bleeding in our study may partly reflect the relatively small sample size and the real-world treatment setting.

The mechanism of ibrutinib-associated bleeding is complex. BTK and Tec-family kinases participate in platelet activation pathways, and their inhibition can impair platelet signaling and aggregation. CLL itself may also be associated with impaired platelet function. Interestingly, platelet function may improve during treatment as the leukemic burden decreases [7].

Diarrhea and gastrointestinal toxicity

Diarrhea is another recognized adverse effect of BTKi therapy. It has been associated, at least in part, with off-target kinase inhibition. A review of BTKi toxicities reported diarrhea in approximately 3-65% of patients receiving ibrutinib and 17-58% of patients receiving acalabrutinib, with severe diarrhea being less common with acalabrutinib [8].

In our study, gastrointestinal adverse events, including nausea and diarrhea, occurred in seven patients (17.0%). Grade 3 diarrhea occurred in one patient (2.4%), who was receiving ibrutinib.

The relatively low frequency of severe diarrhea in our cohort is consistent with the generally manageable gastrointestinal toxicity reported with second-generation BTKi. Nevertheless, severe or persistent diarrhea may affect treatment adherence and may require supportive therapy, temporary treatment interruption, or modification of therapy.

Treatment discontinuation

Treatment discontinuation is an important consideration with continuous BTKi therapy. Real-world data have demonstrated that ibrutinib may be discontinued because of toxicity or disease progression, emphasizing the importance of monitoring patients throughout treatment.

In clinical practice, treatment discontinuation may be influenced by adverse effects, comorbidities, disease progression, financial considerations, and patient preference. In India, these considerations may be particularly important because access to targeted therapy varies across patients and healthcare systems.

Infectious complications

Infectious complications remain an important concern during BTKi therapy. A systematic review by Tillman et al. reported infections of any grade in approximately 56% of patients receiving single-agent ibrutinib and 52% receiving combination therapy, with pneumonia contributing substantially to infectious morbidity [9]. Varughese et al. also described serious infections among patients receiving ibrutinib for lymphoid malignancies [10].

In our cohort, four patients (9.7%) developed lower respiratory tract infections. Although the overall frequency was lower than that reported in some clinical studies, infections remain clinically important in patients with CLL because of underlying disease-associated immune dysfunction and the additional effects of therapy.

Prognostic factors and survival

Prognostic factors remain important in CLL management. Soumerai et al. developed and validated a prognostic risk score for patients with relapsed/refractory CLL receiving targeted therapy or chemoimmunotherapy; the model incorporated β2-microglobulin, hemoglobin, LDH, and time from initiation of the last therapy [11].

In our study, survival differences by age and LDH were not statistically significant. This may be related to the relatively small sample size and limited follow-up period.

Real-world registry data have also demonstrated the importance of cytogenetic and molecular prognostic testing in CLL. Mato et al. reported that FISH/cytogenetic testing provides clinically relevant prognostic information, with abnormalities such as del(17p) and del(11q) associated with less favorable outcomes [12].

Richter transformation

Richter transformation refers to the development of an aggressive lymphoma, most commonly DLBCL, in a patient with CLL [13]. Transformation is associated with an adverse prognosis and requires a change in therapeutic strategy.

In our cohort, Richter transformation occurred in one patient (2.4%). The patient developed DLBCL while receiving ibrutinib and was subsequently treated with R-CHOP-based chemotherapy. This incidence falls within the range reported in the literature, although comparisons should be interpreted cautiously because of differences in patient populations and follow-up duration.

The occurrence of Richter transformation in a patient receiving BTKi therapy emphasizes that BTK inhibition does not eliminate the risk of clonal evolution or transformation. New lymphadenopathy, rapidly enlarging lymph nodes, constitutional symptoms, elevated LDH, or other features suggestive of aggressive disease should prompt evaluation for Richter transformation.

Long-term outcomes with ibrutinib

The RESONATE trial established ibrutinib's efficacy in relapsed/refractory CLL, showing significantly improved progression-free and overall survival compared with ofatumumab.

Similarly, long-term follow-up of the RESONATE-2 study demonstrated durable efficacy of first-line ibrutinib in older patients with CLL. At five years, the estimated progression-free and overall survival rates with ibrutinib were 70% and 83%, respectively [14].

In our study, at a median follow-up of 24 months, progression-free survival was 80%, and overall survival was 95%. These findings are encouraging and demonstrate the effectiveness of BTKi therapy in a real-world Indian population. However, direct comparisons with randomized trials should be made cautiously because our study was retrospective, single-center, and included a relatively small number of patients.

High-risk disease

High-risk molecular abnormalities, particularly del(17p) and TP53 abnormalities, are important determinants of CLL outcomes. Targeted therapies have substantially improved treatment options for these patients.

Acalabrutinib has demonstrated non-inferior progression-free survival compared with ibrutinib in previously treated CLL and has been associated with fewer cardiovascular adverse events, particularly atrial fibrillation, in comparative studies.

Another selective BTK inhibitor, ONO/GS-4059, has demonstrated activity in CLL, providing further evidence supporting the development of more selective BTK inhibition. In a phase 1 study, 24 of 25 evaluable patients with CLL responded to ONO/GS-4059 (96.0%) [15].

Our study was not powered to determine the independent prognostic significance of individual high-risk abnormalities. Larger multicenter Indian studies are needed to clarify the outcomes of patients with del(17p), TP53 abnormalities, and other high-risk molecular features in routine practice.

del(13q)

Deletion 13q14 (del(13q)) is one of the most common cytogenetic abnormalities in CLL and, when present as an isolated abnormality, is generally considered a favorable prognostic marker [16,17].

In our cohort, del(13q) was identified in 15 patients (36.5%) and was associated with longer survival on BTKi therapy. This observation is consistent with the recognized favorable prognostic significance of isolated del(13q). However, because of the limited sample size, this association should be interpreted cautiously.

The prognostic effect of del(13q) is heterogeneous and may depend on factors such as deletion burden and deletion size. Studies have shown that a higher proportion of cells carrying del(13q) may be associated with less favorable outcomes, highlighting the importance of characterizing the deletion rather than treating all del(13q) abnormalities as biologically equivalent [17].

Indian real-world experience

Relatively few published studies evaluate BTKi treatment in Indian patients with CLL [18]. Agarwal et al. reported real-world experience with ibrutinib through a Named Patient Program involving Indian patients with relapsed/refractory CLL and mantle cell lymphoma. In that study, six patients had CLL and two had mantle cell lymphoma; infections were among the reported adverse events.

The present study adds to the limited Indian real-world evidence by including both ibrutinib- and acalabrutinib-treated patients and evaluating treatment outcomes, toxicity, cytogenetic characteristics, and survival.

The healthcare environment in India differs from that of many Western countries in drug accessibility, reimbursement, treatment costs, and the availability of molecular testing. These factors may influence treatment selection, treatment continuity, and outcomes in routine clinical practice.

Strengths and limitations

The principal strength of this study is that it represents real-world experience with BTKi therapy from a tertiary healthcare center in North India. The study includes patients treated in both frontline and subsequent-line settings and evaluates both efficacy and clinically relevant toxicities.

However, several limitations remain. First, the study was retrospective and single-center. Second, the sample size was relatively small, with only 41 patients included in the analysis. Third, follow-up was relatively short, with a median follow-up of 24 months. Fourth, retrospective assessment of adverse events may underestimate low-grade toxicities. Finally, the study was not powered to independently assess the prognostic impact of individual cytogenetic or molecular abnormalities.

Larger prospective, multicenter studies from India are needed to validate these findings and assess long-term outcomes, treatment discontinuation, financial toxicity, and comparative outcomes between different BTKi.

## Conclusions

BTKi have significantly advanced the treatment of CLL, providing effective therapy for both treatment-naïve and relapsed/refractory patients. BTKi therapy is effective and well tolerated in Indian patients with CLL; it is effective across molecular subtypes, is straightforward to use, and is associated with predominantly manageable adverse effects. The CLL treatment landscape in India is changing rapidly. Increasing access to cost-effective generic BTKi and support from social health insurance schemes may improve access to CLL treatment in India. There is a clear shift in patient preference toward BTKi-based regimens for CLL.
